# Book Reviews

**Published:** 1830-01

**Authors:** 


### CRITICAL ANALYSES.

Quæ laudanda forent, et quæ culpanda, vicissim

Illa, prius, cretâ; mox hæc, carbone, *notamus.*—Persius.

*Hospital Facts and Observations, illustrative of the Efficacy of the new Remedies, Strychnia, Brucia, Acetate of Morphia, Veratria, Iodine, &c. in several Morbid Conditions of the System; with a comparative View of the Treatment of Chorea, and some Cases of Diabetes; a Report on the Efficacy of Sulphureous Fumigations in Diseases of the Skin, Chronic Rheumatism, &c.* By James Lomax Bardsley, m.d. Physician to the Manchester Infirmary, Dispensary, Fever Wards, Lunatic Hospital, and Asylum; and Lecturer on the Principles and Practice of Physic, Materia Medica, and Medical Botany.—8vo. pp. 223. Burgess and Hill, London, 1830.

Before we enter upon the analysis of a work, it is our invariable custom first to peruse the whole of it carefully and attentively. We do not, like some "reviewers" whom we could mention, if fraternal courtesy did not forbid the exposure, at once proceed with pen and scissors, to introduce a few flourishing sentences, to cut here and there a page from the author, and then wind up with some general expressions of gratitude, mingled with a little alloy of disappointment. In such a plan of "doing a review," there is, indeed, an attractive safety which is tempting; for, as no positive opinion is hazarded, the judgment of the critic cannot well be impugned. If the author should complain, the critic may easily defend himself, for he has been grateful for his work: if the profession in general should venture to doubt the infallibility of a reviewer, he is again protected, for he was not altogether satisfied with the performance.

We find, from the preliminary examination which we have made of his work, that the task which Dr. BARDSLEY has imposed upon us is not difficult. He has indulged in no speculations, and therefore there is no room for criticism. He has collected a valuable body of practical facts relating to the therapeutic properties of certain remedies which have as yet been but little tried in this country, although their merits have been highly extolled by our continental brethren. We shall submit to our readers the results of Dr. Bardsley's experience, together with some of his most interesting cases.

The work contains cases and observations chiefly derived from the author's practice at the Manchester Infirmary; and, as a proof that this institution affords ample opportunities for the observant physician to enrich his own mind, and add to the practical information of others, it is stated "that, from the 24th of June, 1827, to the 24th of June, 1828, 16,680 of the different classes of in, out, and home patients enjoyed the benefits of this extensive charity." Dr. Bardsley has chiefly availed himself of the great advantages of his situation, by selecting for his inquiries that very important class of substances, the vegetable alkalies, for the purpose of determining their real therapeutic properties. With very laudable, and we regret to say unusual, candour, he relates unsuccessful as well as favorable results attending their use in his hands. Besides the vegetable principles noticed in the work, the author has made trial of *Picrotoxia*, *Delphia*, *Solania*, and *Lupulia*, in several affections; but they have proved useless remedies in disease, and therefore the experiments that have been made with them are not detailed.

The first subject to which our attention is directed is "the Medicinal Properties of Strychnia in Paralysis, with illustrative Cases."

The strychnus (nux vomica) is ranked by Linnæus in the natural order *Luridæ*; and by Jussieu, in his natural arrangement, *Apocineæ*. The tree which affords the vomic nuts of commerce grows abundantly in Ceylon, Malabar, and on the coast of Coromandel. It appears, from the experiments of various physiologists, that the action of nux vomica varies in its effects on different animals; that its

The Buceros (hornbill) feeds upon it, deleterious influence is more rapidly manifested when a portion of it is injected into the pleura, peritoneum, or jugular vein, than when introduced into the vessels remote from the heart, or applied to any of the mucous surfaces of the body; and that its action upon the system is destroyed by previously removing the spinal marrow. In animals destroyed by nux vomica, no marks of inflammation have been found upon dissection. It appears that it does not occasion any organic lesion, but that it has a direct action upon the nervous system, causing death from the asphyxia produced by the immobility of the chest during the violence of the tetanic spasms of the thoracic and abdominal muscles. By careful analysis, Pelletier and Caventou discovered in the vomic nut two vegetable alkalies, *strychnia* and *brucia*. The nux vomica has long been employed as a remedy in many diseases, but its use was almost entirely neglected until Magendie, Delile, and Fouquier called the attention of the profession to its remedial properties. Magendie established the fact, that the nux vomica exerts an especial action on the spinal marrow, and nerves emanating from it, as well as on the muscles which those nerves supply; and hence Fouquier was led to make trial of it in several cases of paralysis of the inferior extremities. Magendie was at the same time engaged in a series of similar experiments with the nux vomica, which afforded additional evidence of its value in paralytic affections. Dr. Cooke used it with varied success. Georget* and Alibert† employed it without much advantage. The early trials of Dr. Bardsley were made with the extract of nux vomica, but, from the uncertainty of this preparation, he relinquished its use in favor of strychnia, whose action is more *certain* and *uniform*.

"Case I. Mary Mitchell, aged thirty years, admitted 28th March, 1824.

"She has entirely lost the power of the left side, with diminished sensibility. Complains also of occasional severe headach, and is liable at times to sudden attacks of vertigo. Her articulation is much impaired. Urine and feces passed involuntarily in bed. Corner of the mouth much drawn to the right side; pulse eighty-six, rather feeble. Countenance pallid. Sleeps ill. The attack occurred about three months ago, shortly after being delivered of twins, and has gradually increased. She attributes her complaint to over fatigue and cold, when far advanced in pregnancy. Has used several remedies, but is ignorant of their nature.—Ordered

* De la Folie, p. 502.

† Nouveaux Elemens de Therapeutique, tome i. p. 438.

five leeches behind each ear, a blister to the nape of the neck, and a dose of the common purging mixture of the house.

"April 1st.—Leeches bled freely, and blister discharged well, with relief to pain in the head. Several copious stools obtained from purgative. To commence with the twelfth of a grain of strychnia, in the form of pill, twice a day.

"4th.—Symptoms unchanged. Strychnia pill to be taken three times in the day.

"7th.—Head remains free from uneasiness. No perceptible effect from alkali.

"10th.—The dose of strychnia to be increased to the eighth part of a grain, three times a day. Bowels regular.

"14th.—The alkali has not as yet occasioned any manifest effect upon the system. The fourth of a grain to be exhibited three times in the day.

"20th.—Has again complained of slight pain in the head, but without vertigo. She states that she experienced yesterday a slight sense of prickling in the paralytic members, which continued for some time after each dose of the pills. No medicine required for bowels. Leeches to be repeated.

"24th.—Pain in the head very trifling since repetition of leeches. To continue.

"27th.—She appears to possess much more feeling in the affected side, as well as increased power over the paralysed muscles. Makes no complaint of pain in the head this morning.—Half a grain of strychnia to be taken twice in the day.

"30th.—On the second day after the exhibition of the alkali in this proportion, the patient experienced smart convulsive twitchings of the muscles of the diseased side. They are now present.

"May 3d.—She can move the paralytic limbs much better, and begins to feel conscious when the bladder and rectum are evacuated.—To take one grain of strychnia twice a day.

"6th.—Head became affected with stupor and vertigo, and rigid contractions of the muscles of both sides of the body supervened to the employment of the third dose of the alkali, in the proportion noticed in the report of the 3d. This quantity, however, was repeated yesterday and also this morning, and has been unattended by the former severe effects of the medicine. The patient has regained a considerable degree of power over the leg and arm, and the tone of the sphincters of the bladder and rectum is much restored. Not deeming it prudent to increase the dose of the alkali, she was directed to continue the one-grain dose twice in the day.

"14th.—This dose now occasions no inconvenience. To continue the strychnia in doses of half a grain three times daily.

"17th.—She is now much better; can hold a cup to her mouth when she wishes to drink, and also raise her left leg from the bed. She sits up during the day, and regularly asks for the bedpan when she requires it. Speech more distinct.—Pills to be continued.

"20th.—Continues to improve. To persevere with the pills.

"28th.—From the date of the last report up to the present period, her amendment has been rapid, for she now not only supports herself in the upright posture with the aid of crutches, but even walks with them from one bed to the other. Her strength, articulation, and general health, are much improved; appetite keen." (P. 7.)

This woman left the hospital at her own request, greatly relieved, and in about two months after her discharge she had recovered the perfect use of the paralytic members, and could attend to the affairs of her family as well as at any former period of her life.

Twenty-three cases are related in which the strychnia was employed in paralysis, and Dr. Bardsley concludes with the following observations:

"The above recital of cases is, I conceive, amply sufficient to show that the strychnia exerts a very powerful influence upon the system, and that it is entitled to rank as a valuable remedy in the list of articles belonging to the materia medica. It was employed in some of the cases of paralysis with no benefit, in others with only partial advantage, but in the majority with complete success: hence it may with justice be considered an efficacious, though not a certain, remedy in this affection. Indeed, it is almost in vain to expect a cure from any of the means supplied by our art, when cerebral disorganization has occurred. 'Whenever (as my relative Dr. Bardsley, when speaking of the employment of galvanism in paralysis, has observed,) paralysis arises from tumors compressing the substance of the brain, or from a diseased alteration in its mass and structure, or from extravasation of a fluid in such a state or degree as will not admit of its absorption, it will be readily admitted that no benefit is to be expected from the employment of galvanism, or perhaps any other remedy yet discovered.'* It is in such cases of paralysis as seem to arise from diminished nervous excitement, that the strychnia is particularly indicated.† It may be stated here, as a rule of guidance, that whenever hemiplegia supervenes to an apoplectic seizure in persons of a plethoric habit, it is proper to employ bleeding, purging, and the ordinary antiphlogistic treatment, before resorting to the use of the strychnia. When the vessels of the head have been freely unloaded,

* "Medical Reports, p. 215.

† "My learned friend, Dr. Milligan, (to whom I take this opportunity of publicly acknowledging my obligations for the encouragement he has kindly given me in my present undertaking,) has expressed to me his doubts of the correctness of this doctrine; for he states that there are several cases on record where the person has recovered the use of his limbs under such disorganization. (See, e. g. in Abercrombie's Path. of the Brain, p. 263, 4, an instance of a man recovering three or four times.) Chardel, Louis, and others, have collected similar examples.

and the quantity and force of the circulating fluid diminished by the above means, there can be little objection to a cautious and prudent trial of this remedy. Generally speaking, the strychnia is likely to prove more serviceable in paraplegia, unconnected with spinal disease, than in hemiplegia; though I feel confident that it will not unfrequently be found an important remedial agent even in hemiplegic paralysis. My experience with this substance has been confined entirely to adults, for I have not deemed it prudent to administer so powerful a medicine to children, nor should I advise the attempt. In the above cases, I have endeavoured, in as far as the circumstances of each would allow, to observe simplicity of prescription, being convinced that it is next to impossible to decide upon the remedial virtues of any individual substance when the practice is complex. In several instances it will be found that the strychnia alone was exhibited, (excepting, perhaps, an occasional dose of aperient medicine,) and its efficacy was most decided. The first effects of the alkali, in every case, were convulsive twitchings of the paralytic members, and no benefit was derived until this condition of the parts had been produced, and continued for some time. In more than one of the instances of paralysis treated by Dr. Manson with iodine, the exhibition of that remedy seems to have been attended with a similar result. Thus, in the case of Elizabeth Spooner, we have the following report of the 23d February: 'I find that, on the 16th instant, she was very much troubled with twitchings and involuntary shaking of the left hand, previous to its being in a great measure restored. She can now open and shut the hand freely, and raise it as high as the crown of the head.' In the case, too, of Charles Battersby, the report of the 2d November states that, 'he has more twitching and pain in the paralytic parts since the 31st ultimo, than hitherto; has more feeling in them; and has been able, for the first time, to raise the left thigh to a right angle with the body.'* In Dr. Alderson's experiments, too, with the rhus toxicodendron in paralysis, twitchings and tingling sensations in the paralytic members appear to have been the first steps towards recovery. Strychnia possesses an advantage over some other internal remedies; for it does not impair the energy of the stomach, but is rather serviceable in promoting appetite and digestion. This was very evident in several of the cases I have detailed. When it is decided upon to administer the strychnia, it is proper to commence with a proportion not greater than the eighth of a grain twice in the day. This quantity may be gradually increased to the sixth, fourth, or even half a grain, at the same intervals. The first effects of the remedy must be carefully noticed, and, should symptoms of an unpleasant nature occur, it must be immediately suspended. After a short time, its use may be resumed, and continued in slowly augmented doses, so long as the judgment of the practitioner may

* Manson on Iodine, p. 125, 136.

approve. By attention to these points, although no benefit may accrue from the strychnia, we may be sure that no injury will attend its exhibition. I have generally administered it as a pill, with a little conserve of roses, and in this form it has been most agreeable to the generality of patients." (P. 38.)

In the second case, the dose of the alkali was increased to half a grain three times every day, with strict orders that its effects might be watched. After the fourth dose, the patient was suddenly seized with vertigo, vomiting, sinking of the pulse, uneasiness about the præcordia, difficult respiration, rigid contraction of the paralysed muscles, and copious perspiration, particularly over the head and face. These symptoms continued for some time, but yielded to the liberal administration of active stimulants, as brandy and volatile salt. The remedy was then discontinued for one day.

*Strychnia in cases of Chronic Diarrhœa.* Numerous instances of chronic diarrhœa occur among the out-patients of the Manchester Infirmary, some of which resist almost every variety of treatment adopted for their cure. In such cases Dr. Bardsley tried the strychnia, and "it proved a *safe* and *effectual* remedy." Six cases are detailed of chronic diarrhœa cured by strychnia.

"I do not consider the strychnia a suitable remedy in those instances of diarrhœa which depend upon an evident inflammatory condition of the mucous membrane of the intestines, but I more particularly recommend it in cases of a chronic kind, occurring in persons somewhat advanced in life, and of feeble constitution.

"It may be proper to state, that it is not my wish to propose strychnia as a remedy in this form of diarrhœa, to the exclusion of those sedative, diaphoretic, tonic, and astringent medicines, whose utility has been confirmed by long experience; but merely as deserving of attention when they have been unsuccessfully tried.

"The strychnia also exerts a beneficial action upon the stomach, by restoring its tone and powers of digestion.* My intelligent friend, the late Dr. Dewar, after noticing the employment of opium, catechu, kino, alum, ipecacuanha, sulphate of zinc, chalk, and hæmatoxylon, in diarrhœa, remarks that 'no one of those astringents has been found remarkably preferable to the rest: they are all of them often effectual, and all of them are liable to fail.'† It is in such obstinate cases as these that the aid of strychnia may be advantageously solicited." (P. 49.)

* "It is probable that this is the manner in which it proves useful in chronic diarrhœa, for its effects are too slow to be supposed to operate in the way of other remedies in that disease.

† Observations on Diarrhœa and Dysentery, p. 60 and 6 .

Without entering upon an inquiry into the *causes* and *consequences* of Amenorrhœa, the author next proceeds to illustrate the successful administration of strychnia in his hands in some instances of *suppressed* menstruation. Four cases are described at length, and a tabular account of eight others is given. The result was very satisfactory, and amply sufficient to show the remedial virtue of strychnia in some instances of amenorrhœa.

"This is in a great measure owing to the power which the alkali possesses of stimulating the vessels of the uterus, and of improving the tone and vigor of the system. I have remarked a confirmation of this fact in two or three instances of females, with whom the menses have returned during the use of the strychnia, even after they had disappeared for more than a year and a half. I should advise the conjoint exhibition of mild laxatives with the alkali in this affection, when the bowels, as is most commonly the case, are constipated." (P. 57.)

Brucia, the other vegetable alkali detected in the nux vomica by Pelletier and Caventou, has also been employed by Dr. B. in paralysis. These chemists likewise procured it from the bark of the pseudo-angustura (false angustura,) which grows in South America. Dr. Kinglake, in a paper published in the seventeenth volume of our Journal, has alluded to a poisonous species of angustura bark having been sold by the druggists at Taunton in 1807, which, in several instances, produced the most distressing effects. It is highly probable, Dr. B. thinks, that the noxious kind of bark noticed by Dr. Kinglake was obtained from the pseudo-angustura, and introduced into the market instead of the Bonplandia trifoliata. It appears, from the experiments of Andral, that one quarter of a grain of pure strychnia equals in energy six grains of brucia. Ten cases have been selected by the author from some others, as affording the best evidence of the remedial value of brucia in paralysis. He thus sums up his opinion respecting this remedy:

"The results of my trials with brucia lead me to recommend it as a valuable medicine in that affection. The action of this alkali upon the system is, as before observed, analogous to that of strychnia, but less powerful; hence it is a preferable remedy in paralytic attacks, accompanied with much cerebral disturbance. When the brucia is employed, it is prudent to commence with a proportion not greater than a grain, taken twice daily, which may be cautiously advanced to the exhibition of two grains, three or four times in the day. In the case of Andrews, which I have detailed, it appears that he was incapable of taking a two-grain pill five times daily, without experiencing symptoms of an unpleasant nature. I have noticed the same result, too, in some other instances. With respect to the length of time necessary to give the brucia a fair trial in paralytic affections, I should say, from my experience with this remedy, that, unless a marked advantage accrue from its use in the course of five or six weeks, it may be very properly laid aside." (P. 75.)

Acetate of Morphia *in some instances of painful Affection of the Stomach.*—Morphia *alone* has no action upon the system, on account of its insolubility. It has, therefore, been combined with acids, to form soluble salts. Hence we have the acetate, the sulphate, and hydrochlorate of morphia. Magendie has employed these salts as remedies in disease, and states that he derived from them all the advantages of opium, without any of its inconveniences.* In a severe case of spasmodic affection of the stomach and bowels, occasioned by an incautious use of iodine, Dr. Gairdner states, "that the young lady's life was saved by a quarter of a grain of acetate of morphium given every half-hour. Every other form of opium was tried without effect: they were not even retained an instant on the stomach. The acetate of morphium alone could be taken, and it effectually restrained the disease, which must otherwise have very soon terminated the life of the patient."† In other cases, however, this medicine did not answer his expectations. The acetate of morphia has been successfully used as an external remedy in tetanus, hooping cough, &c.‡

Dr. Bardsley relates several cases in which this remedy was administered in painful affections of the stomach, and in scirrhus and induration of the uterus. The following instance of its good effects in painful menstruation is interesting:

"A female, thirty years of age, was subject to the most excruciating agony at each return of the menstrual discharge. Her suffering was so great, that she generally remained insensible for some hours. Her general health seemed to be good, and her bowels were regular. She had been under medical care both in Liverpool and Chester, but had not derived any relief from the remedies which had been employed. At the time of her applying to me, she expected the catamenia in about ten days. I directed her to take a quarter of a grain of the acetate of morphia on the first accession of pain, and to continue this proportion every half-hour, whilst it was violent. She called upon me in the course of

* Nouveau Journ. de Med. 1818, *and* Formulaire pour la Preparation et l'Emploi de plusieurs nouveaux Medicamens.

† Essay on the Effects of Iodine, p. 24.

‡ Archives Generales, Octobre 1829.

a fortnight, and stated that the second dose afforded her very great relief, without suppressing the menstrual discharge, which was natural both in quantity and appearance. She now takes the morphia regularly at every monthly period, and with like advantage." (P. 99.)

Six cases of neuralgia are adduced to show the utility of the acetate of morphia. Upon the general effects of this remedy, Dr. Bardsley remarks,

"It must be allowed that morphia has not always answered the intentions with which it has been employed, but the proportion of favorable cases has been considerable, compared with those in which it has failed. I have never witnessed any pernicious consequences from a prudent use of the morphia. I am led to recommend the acetate of morphia in preference to opium, from a conviction that its efficacy may be equally relied upon, whilst its administration will be unattended by the distressing headach, excessive constipation, and other unpleasant symptoms, which that drug in large doses mostly induces. It appears to be the chief advantage of morphia, that it may be employed in those cases in which it is desirable to obtain a narcotic effect, and at the same time of the first importance to avoid constipation. It is proper to unload the bowels before commencing with the acetate, so as to give the remedy a fair chance. It is prudent to commence with not more than a quarter of a grain, which may be gradually increased to half a grain, a grain, or two grains, according to the urgency of the symptoms and the effect produced. These doses are applicable to adults. I have mostly prescribed it in the form of pill, which seems to answer very well." (P. 107.)

We prefer administering this potent remedy in a liquid form.* A minute dose may thus be more accurately given, and, as far as our own observation extends, the solution is more to be depended upon in its effects. Our experience of the superior advantages of the acetate of morphia over other opiates, coincides with that of Dr. Bardsley.

In dropsy and chronic rheumatism, Dr. B. has used the Veratria and Colchicum Autumnale with much advantage. The alkaline substance named veratria was first obtained from the Veratrum *sabadilla* by those indefatigable chemists, Pelletier and Caventou. They afterwards detected it in the Veratrum album and Colchicum autumnale. In twenty-four cases of chronic rheumatism treated with veratria, ten were relieved, seven cured, and seven were not relieved. In the same number of cases of the same disease treated with colchicum, eleven were relieved, seven cured, and six remained unrelieved.

* Mr. Garden, of Oxford street, keeps a very convenient solution of the acetate of morphia. Six drops contain one grain of the substance.—Ed.

"*Remarks.* In the preceding cases, the veratria and colchicum autumnale were exhibited with very similar results. The action of the two substances upon the system was also analogous, for both produced frequent watery evacuations from the bowels. I have given the veratria and colchicum rather extensively at our hospital, both in acute and chronic rheumatism, but it must be allowed that they have often failed in affording any permanent benefit. The late Mr. Haden seems to have used the colchicum as a substitute for bleeding, in inflammatory affections, with almost uniform success.* I have given this remedy in the manner pointed out by that author, but with very different results. In more than one instance of acute rheumatism, I have had to regret the neglect of the lancet. Such practice is pregnant with danger. 'It is seldom,' the reviewer of Mr. Haden's work justly observes, 'that the very severe form of fever which accompanies acute rheumatism, can be subdued by any remedy short of copious bloodletting: indeed, we should not wish to see a smart attack allowed to proceed, when we are possessed of means so efficient to arrest its progress.'†

"The utility of colchicum in gout is confirmed by general experience. In most of the cases of that disease in which I have seen this remedy employed, little or no advantage was procured until the occurrence of purging. My esteemed colleague Mr. Simmons, who has been a martyr to the gout for several years, informs me that he has obtained from the wine of the seeds of colchicum a relief in the paroxysms of that harassing malady, which he had in vain sought for in any other remedy. With that gentleman, the benefit from that medicine seems to depend upon its purgative operation. In some cases, however, it must be allowed that the colchicum removes the paroxysm of gout without any sensible operation of any kind. In proof of this fact, Mr. Want has adduced the case of the late distinguished Sir Joseph Banks.‡ I am also acquainted with a highly respected clergyman, in this neighbourhood, upon whom the colchicum produces no sensible effect.

"In the exhibition both of veratria and colchicum in moderate doses, I have always observed the pulse in a short time to become slower and depressed; and, if the proportion has been much or rapidly increased, distressing nausea or vomiting, and purging, have been excited. It is necessary carefully to watch the effects of these remedies, since, in an over dose, they are apt to occasion an alarming and sometimes fatal train of symptoms. I have generally commenced with the fourth of a grain of veratria, gradually increasing the dose to half a grain thrice daily, or a grain twice in the day; and with fifteen or twenty minims of the wine of the

* See Practical Observations on the Colchicum Autumnale, &c.
† Edinburgh Medical and Surgical Journal, vol. xvii. p. 452.
‡ Vide London Medical and Physical Journal, vol. xxxii. p. 202.

seeds of colchicum, slowly increased to twenty-five or thirty, at the same intervals. The stomach will be found but rarely to retain more than the last-named quantities of these medicines; and patients hesitate to continue their use for any length of time, when frequent nausea and vomiting are induced." (P. 117.)

We have next a few brief observations on the virtues of IODINE in bronchocele, scrofula, &c. Dr. Bardsley thinks that occasional advantage may be derived from this remedy in some instances of dropsical effusion, where there is reason to suspect obstruction to the free return of the blood. It is more particularly in ascites depending upon some enlargement of the liver, or the presence of steatomatous tumors in the abdomen, that iodine is likely to afford some chance of relief. In the whole of his trials with iodine, the author has employed internally a solution of hydriodate of potass, in the proportion of half a drachm to an ounce of distilled water; and, as an external application, two scruples of the salt to an ounce of axunge. Dose of the former, ten drops twice and thrice a day, gradually increased to twenty at the same intervals; and of the latter, a drachm has been directed to be rubbed in over the affected part night and morning. Much caution is requisite in the use of this powerful medicine.*

Dr. Bardsley's experience of CINCHONIA and the SULPHATE of QUINIA, confirms the opinions commonly entertained by the profession.

GENTIANA he has found useful in dyspepsia with irritability of stomach. The form of pill is preferred to the tincture.

In 1817, Pelletier and Magendie published the results of their analysis of the roots of the several species of ipecacuanha, from which it appears that each variety owes its emetic property to a peculiar principle named EMETINA. They inform us that they derived from it all the ordinary effects of ipecacuanha, without its disagreeable odour or taste, and recommend it in chronic pulmonary catarrh and protracted diarrhœa. Dr. Bardsley says,

"In the dose of five grains, dissolved in two or three ounces of rose water, it has proved an active emetic. In the proportion of half a grain every five hours, it has acted as a mild diaphoretic; and, in the dose of a fourth of a grain every three hours, as an expectorant. It has produced these effects with great certainty. In some instances of dysentery, chronic diarrhœa, and chronic

* Even the application of the iodine ointment may produce injurious effects. A case in point is related in our November Number, p. 471.—ED.

pulmonary catarrh, I have derived from the emetina, in combination with a small proportion of opium, much benefit.* I have generally used it in the form of a pill, with a small quantity of aromatic confection. My trials with emetina do not lead me to recommend it as a substitute for the ordinary powder of ipecacuanha, except as a remedy for children, and in certain cases of idiosyncrasy, in which the effluvium of that drug is found to occasion highly pernicious effects. Several examples of this kind are upon record."† (P. 149.)

In the next section, a comparative view of the remedies in Chorea is given. Dr. B. has found that this disease is both more *certainly* and more *speedily* removed by purgatives and antispasmodics, than by either of them exhibited *singly*.

Some cases of Diabetes are reported, and the efficacy of the conjoint employment of *animal diet, opium*, and the *warm bath*, appears to be established by them.

"As to the perfect cure of diabetes, it is necessary to speak with much caution; for, like some other formidable diseases, it is mostly capable of being relieved only, and not effectually removed. 'Within these last six or seven years,' says Dr. Prout, 'nearly twenty cases of diabetes have fallen, more or less, under my observation; and among these I have never, but in one instance, and in that for a very short time only, seen the urine of a diabetic patient rendered quite natural.'" (P. 191.)

The volume concludes with a report on the utility of Sulphureous Fumigations in some diseases of the skin, chronic rheumatism, diabetes, &c., with some general observations on the treatment of certain cutaneous affections.

Dr. Bardsley's work will be consulted with much advantage by every practical physician. His reports bear internal evidence of being most faithfully recorded, and they must be esteemed a valuable addition to our information respecting the remedial powers of the very important medicines to which he has directed our attention

It would have extended the present article to too great a length had we extracted more of the cases; but we shall occasionally select the most important of them, and give them under the head of Hospital Reports.

* It may be proper to observe, that in these cases the impure or coloured emetina was employed.

† Vide Medical and Physical Journal, vol. xxiii.

*Notions of the Nature of Fever and of Nervous Action.* By William Forrester Bow, m.d. Senior Physician to the Alnwick Dispensary, and formerly Assistant Surgeon of his Majesty's 27th and 77th Regiments.—8vo. pp. 100. Longman, London, 1829.

Our notions respecting the nature of fever are still beset with too many perplexities to justify us in passing over with neglect the contribution of any respectable writer upon so important a subject. A part of the contents of the present volume has already appeared in the pages of our Journal; but since the author favored us with his original papers upon the subject, he has found it requisite to modify some opinions he formerly entertained.

Dr. Bow first communicated his opinions to a friend, and he has not deemed it necessary to alter the style: his essay is therefore divided into five letters, the substance of which we shall lay before our readers.

"Those," says Dr. B. "who have lost hope of ever becoming intimate with the now mysterious essence of fever, content themselves with the thought that a rational and successful plan of treatment may be formed, independent of the knowledge of its proximate cause. In this opinion, however, I cannot coincide; for, although we be successful in our practice, and therefore call our plan rational, we but meet a symptom, as it appears, with the remedies with which experience has armed us, which chance perhaps had first directed, and we anticipate another, which experience teaches us to look for: but is it not humiliating to acknowledge we know not what gives birth to the one, nor how the action we induce operates in preventing the other?"

One physiological fact, now acknowledged by all, will, in the opinion of the author, go far to unravel the mystery, and assist us in forming a theory: viz. that the function of secretion is at least under the influence of the nervous system. This is undeniable; but, to him, the facts and arguments of those who refer secretion altogether to nervous power, seem convincing. Some of the objections in opposition to this theory are briefly adverted to.

"There is nothing, it is said, in the ramifications of the vessels composing a gland which can in any way account for the changes in the blood out of which the secretions arise, and therefore it was naturally enough supposed that secretion is effected through the instrumentality of the nerves. In support of this view, Sir Everard

Home observes, 'that in fishes which are capable of secreting the electrical fluid, the nerves connected with the electrical organs exceed those that go to all the other parts of the fish, in the proportion of twenty to one.' 'And, in confirmation of this view of the subject, it may be remarked, that there are no parts of the body more manifestly affected, and few so much so, as the secretory organs, by mental emotion.'"*

In the opinion of Dr. Good, these facts seem to prove that the secretory organs are chiefly influenced by the sensorial system; yet he remarks, that Haller long ago observed that the larger branches of the nerves seldom enter them, and seem purposely to avoid them; the secernent glands have little sensibility, and the secretions of plants, which have no nervous system, are as abundant and diversified, and as wonderful in every respect, as those of animals. To this statement Dr. Bow replies, that

"The motor nerves, which can have no necessary connexion with the secernent glands, do avoid them, and perhaps are purposely and wisely made to do so: and, if the glands possess but little sensibility, although largely supplied with twigs of small nerves, it is evident that all these twigs are not sentient nerves, and that they must proceed from some other source than do the motor and sentient nerves.

"These small twigs of nerves are derived from the great sympathetic, branches of which have been traced to every gland and secreting surface; a fact which alone goes far to prove that the sympathetic nerve is instrumental to the production of the secretions." (P. 4.)

"Secretion is a function which cannot, even in part, be destroyed without endangering life; yet it is denied that that process is performed through nervous agency, although we trace nerves proceeding to the organs of secretion, knowing at the same time that they are nerves neither of sensation nor of voluntary motion." (P. 5.)

Dr. Bostock's principal objection to the nervous hypothesis of secretion, and which he brings forward as a direct argument against it, is, that secretion appeared to be produced in fœtuses which had no nervous system. Dr. Bow has had no opportunity of examining the cases cited; "but, admitting that in all of them the brain, cerebellum, and spinal marrow were wanting, it does not follow there was no nervous system; for, in similar cases, the sympathetic system of nerves has been found perfect." In the case related by Dr. Clarke†, the mola, or imperfect fœtus, is

* Good's Study of Medicine.

† Phil. Trans. vol. 83, p. 154.

stated to have had neither heart nor lungs; neither brain, spinal marrow, nor nerves.

We cannot conceive it possible that the sympathetic system of nerves can be perfect without spinal nerves, from whence that system naturally derives so many fibrils. With respect to secretion, we believe that it can only be considered as *under the influence* of the nervous system. There are many facts on record which prove that secretion may take place independently of any nervous agency. Upon this subject better evidence will not be required than the experiments instituted by Mr. Mayo. "No special organization appears necessary for nutritive secretion, or for the separation from the blood of the elements by which the body grows." "When the fifth pair of nerves had been divided upon the petrous portion of the temporal line in a rabbit, upon breaking off the crown of an incisor tooth, I found the part reproduced as rapidly as in an animal in which the nerves were entire."* Again: "Functional secretion is remarkably under the influence of the nerves." —"Yet I found, upon cutting the nerves of the kidney in a dog, that, in half an hour afterwards, a quantity of urine had accumulated in the pelvis of the kidney, and in the ureter, which had been tied."†

Mr. Burns, of Glasgow, maintained the doctrine that, when vital action is increased in one part, it is diminished elsewhere; "but it seems," observes Dr. Bow, "to have been so much disregarded as to be now of little or no use in physiology or pathology; yet it strikes me that, with the assistance of this law, may be explained many phenomena which have been *elucidated* by referring them to the enigmatical laws of sympathy." Upon this principle the cold creeping sensations on the surface of the body, so frequently felt after a hearty meal, are said not to arise from any particular sympathy between the stomach and the skin, "but because the former, having occasion for an additional supply of nervous influence, above what the spleen can furnish, receives it at the expense of all other parts."

With the exception of the hypothesis which gives to the spleen the function of occasionally imparting nervous influence to the stomach, the doctrine contained in the above sentence may be safely admitted.

Continuing the same train of argument, the author states,

* Outlines of Human Physiology, by HERBERT MAYO, F.R.S. &c. p. 117, second edition.

† Ibid. p. 121.

"If, to the healthy subject, we administer a brisk cathartic, we thereby cause a determination of nervous influence greater than natural to the gut; consequently, during the operation of the medicine, there will be felt a greater or less degree of muscular inability and of coldness to the surface; feelings plainly arising from deficiency of nervous influence, owing to the demand for it elsewhere. But if we administer a moderate dose of opium, we diminish the nervous action of the stomach, and then every other part of the body receives a greater than usual force of nervous influence: hence the hilarity of mind and increased warmth of the surface which it produces." (P. 22.)

If the cause of the preternatural determination to any point be sufficiently powerful, the excitement will be such as to constitute inflammation, and general contractility, and secretion will then be so much impaired as to give rise to sympathetic fever.

Dr. Bow is of opinion that "all the primary symptoms of fever lead the unprejudiced observer to pronounce them to be the effects of diminished energy of the nervous system." The remote causes are said to be certain sedative powers, which impair the energy of the brain, and the first symptoms are just what might *a priori* be expected: listlessness, dejection of spirits, and muscular debility, and an imperfect state of the products of secretion.

"In the first stage of fever, the secretions must be regarded as deficient not only in quantity, but also in quality: and if so, they can only be looked on as foreign matter, which, after a time, must irritate the nervous extremities it comes in contact with. It is this stimulation which, in my humble opinion, determines the hot stage. * * This irritation I also believe to be the cause of the unnatural arterial action, which is one of the characteristics of the second stage." (P. 43-7.)

We observe that when the sentient nerves of a part are irritated, the smaller blood-vessels of the part lose their contractility in a degree proportionate to the excitement of these nerves, and consequently admit of distention; and also that the larger vessels leading to the part appear to be in a state of *excitement.*

"From what we do observe, then, in a part when its sentient nerves are excited, we may infer the condition of the whole arterial system, when all the sentient nerves of the body, and particularly those of the skin, are in a state of excitement. It is plain that there must be an arterial plethora, whereas in the first stage there is venous congestion. And it is equally plain that such a state cannot subsist if the irritability or contractility of the arteries in general be equal to what it is in health; that is, if by these terms be meant the power by which muscular fibre contracts." (P. 47.)

The cold affusion, Dr. Bow believes, acts in arresting the progress of fever by exerting a sedative influence over the sentient nerves: thus the nervous influence which had been elicited towards the surface is set at liberty, to be attracted by the organs which laboured from deficiency. The discrepant opinions as to the *modus operandi* of cold are considered to arise from a want of distinction between its primary action and secondary effect.

"Cold acts sedatively: by producing torpor of the nerves to which it is applied, it renders them insensible to the impressions which naturally elicit nervous influence to their extremities; this is its primary action. Cold acts excitingly: by producing torpor of the nerves to which it is applied, it for the time renders them useless as instruments of transmission of nervous influence; therefore, that force of influence which otherwise would have been transmitted towards their extremities, is directed through other nerves, increasing their power of action: this is its secondary effect, or the effect of its primary action." (P. 51.)

By supposing that the cold affusion acted on the principle of abstracting caloric, Dr. Currie, in the opinion of our author, was led to attach too much importance to febrile heat.

"When cold is applied to the living structure, the sensation produced is not the effect of the abstraction of caloric, so much as it is the effect of repression of the power generating the heat of the part; and this is proved by the fact that, although the abstraction of caloric is always attended with a sensation of cold, we experience sensations of cold independently of the abstraction of caloric, and which can only be referred to diminished nervous action in the part where the sensation is felt. Whether animal heat, therefore, be a product of nervous action, or a principle disengaged during the chemical changes effected by nervous influence, sensations of cold will follow the application of whatever has the power to impair nervous action." (P. 56.)

To show that opium has this effect, some experiments are referred to, which were made by Mr. Ward,* nearly thirty years ago, for the purpose of ascertaining the effect upon the pulse of opium applied externally. Dr. Bow does not mean to deny that cold water does abstract caloric from the surface of the body; but he maintains that it is not owing to such abstraction, nor to the *impression* on the *sensations*, that fever is arrested by cold affusions.

The perusal of Mr. Ward's cases in the early volumes of our Journal, first led Dr. Bow to the employment of

* London Medical and Physical Journal, vol. i. et seq.

opium externally in fevers. Although the author differs from Mr. W. as to the *modus operandi* of opium, he feels indebted to him for the faithful relation of its visible effects, which induced him to adopt a practice productive of the happiest results.

"I know not why the practice of exhibiting opium externally in fevers is not more generally adopted, unless indeed it be the idea that it only acts on the system after having gained the circulation. Those who favor the doctrine of absorption may think that the easiest mode of administering it is the best, and that the marked difference in its effects may rest more on fancy than on truth. To such I would say, give it a trial; and I am convinced that if they fail to quench a febrile thirst by every other means, they will succeed with an opiate liniment. By it they will impart softness and moisture to a tongue having more the appearance of horn than of any thing else; they will promote gastric secretion; they will calm delirium; in fact, they will benefit every organ labouring under the deprivation of nervous influence: and this, simply, by diminishing the sensibility of the nerves of the surface." (P. 69.)

The cold affusion cannot, without danger, be employed after the third or fourth day of the second stage of fever; for then the energy of the nervous system begins to decline, and therefore the patient might not recover from the shock which the sudden and extensive application of cold water imparts.

"But in opium we have a remedy not less powerful in effecting all that can be desired from cold: it acts on the same principle, and for this reason is well adapted as a substitute for it in the after periods of the disease. When its application does not decidedly check the fever, it mitigates the symptoms, and evidently shortens its course. It was once remarked to me by the mother of a patient of mine, that her son did not ask for drink during the night when the liniment was used at bedtime; since which, I have found it to be the case very generally." (P. 71.)

We are by no means inclined to admit the doctrine of Broussais relative to fever to its full extent, but at the same time we are not prepared "to think it rests on a mere illusion, rendered the more deceptive by the intellectual labours of one pre-eminently endowed."

Dr. Bow has not been present at any dissection where ulceration of the mucous and muscular coats of the intestines has been detected after death from fever; nor has he seen a more accurate description of it than that by Dr. Hewett, in our Journal for August and September 1826. The cases there given, he thinks, prove disorganization from want of action, rather than ulceration consequent to inflammation.

"If this disorganization of the mucous coat of the intestine be the result of diminished nervous action, I suspect it may be prevented by the external use of opium in the second stage of fever. I am in the daily habit of employing it in this manner, with the intention of diverting nervous influence from the surface; and as I must declare my astonishment at the rapid recovery of my patients, since my practice has been modified by the views I entertain, may I not believe that, by determining nervous influence to internal organs, I have obviated the tendency to this morbid process, which sometimes is an effect, but never the cause, of the common fever of this country?" (P. 74.)

Dr. Armstrong viewed fever as an affection of excitement from the first, and he considered the nervous symptoms as secondary to vascular disorder. Dr. Bow admits

"That some of the nervous appearances of the first stage do depend on vascular disorder, cannot be contradicted; for we know that the nervous and sanguiferous systems are dependent on each other, and that whatever directly affects the one must indirectly influence the other. Dr. Armstrong ascribes the debility of the first stage to the preternatural accumulations of blood in the vessels about the head, heart, liver, and other internal parts; and doubtless such accumulations must have the effect of increasing the debility. But it is difficult to conceive how retrocession of blood from the surface, and internal accumulations, can take place unless as an effect of nervous disorder. Besides, the debility of fever frequently follows the febrific impression so instantaneously as to give no time for any such accumulations; and therefore it is more likely that the cause of the congestion of blood in the larger vessels is, at the same time, the cause of the debility; which is impaired energy of the nervous system. However much the internal accumulations of blood may increase the debility, they cannot be the cause of it; for, when reaction takes place, these accumulations are removed, and still the debility continues." (P. 80.)

In the last letter, some experiments made by the author are related, in support of his opinion of the *modus operandi* of opium, when externally applied.

Dr. Bow agrees with the generally received opinion, that the contractility of the blood-vessels of an inflamed part is lessened. We perfectly agree with him, "that those who support the contrary doctrine entertain erroneous ideas as to the nature of the action of remedies employed in the cure." The application of an opiate liniment is strongly recommended in many of those bronchial affections so frequent after measles, and for which general and local bleeding are much too indiscriminately employed.

*The First Lines of Botany.* By J. S. Forsyth, Surgeon; author of the "New London Medical and Surgical Dictionary," &c.

Seeing this "Primer of the Linnæan System" in the hands of our young friends, we were tempted to scrape acquaintance with a work which professes to give "a simplified introduction to a knowledge of the vegetable kingdom, including the structure, functions, and phenomena, natural and chemical, of plants," in 184 duodecimo pages. This, thought we, is just the book for the hard-fagging, ill-used student, who is compelled to learn so much in so little time, after five years spent in learning nothing: and our predilection became still further strengthened, when, on opening the magic volume, which promised to reveal the mysteries of Flora, and naturalize its votaries as denizens of the vegetable reign, we found some *curiously-executed* coloured figures, (with the names providentially attached thereto,) for the fewness of which an excuse is proffered, on the score that it is better to refer "the student to objects within his reach, than to abstract his conceptions by indifferently executed engravings, known only to represent some natural object by the precaution taken to have the name of the individual attached to *it;*" on the same principle that the sign-painter of Brentford, to quiet the scruples of his townsmen, obligingly wrote under his work "This is the red lion," as many mistook it for a dun cow. On inspecting the plates, however, our admiration of the philosopher became blended with respect for the saint, who, even whilst expounding the oracles of nature, allowed not his zeal to infringe the injunctions of the Decalogue, by making the graven images like any thing either in heaven or on earth.

A short preface (thanks to the author!) informs us that "the baby system of education, as old as Adam, called the 'interrogative,' has nothing to recommend it, not even simplification of ideas." "To children of tender age, indeed, it may possess some advantages;" "but where no distinctions are drawn between an infantile and a mature mind, the idea is as ridiculous as absurd." "Another method," continues our author, "if any thing, more objectionable, is that which is thrust forward in the conversational or epistolary style, by people indifferently acquainted with the sciences they would thereby promulgate." For our part, in our ignorance, we thought that the epistolary and conversational styles were very different from each other, and that the latter more resembled questions and answers, (in which this book abounds, notwithstanding the exorcism,) than letter-writing: but, whatever it may be, we are told that, "even in the best hands, there is something in this method at which the unassuming, industrious, and inquisitive mind recoils; and more particularly when forced, as it were, to draw, through such equivocal channels, upon the fictitious correspondence of some garrulous old woman or pedantic spinster, for the higher order of elementary knowledge."—Hear this, ye authors of "Conversations on Botany," "Letters on Botany," &c.! Hear this, ye shades of Wakefield, Martyn, and Rousseau!! But we are wrong in dallying with the "Preface," while her less querimonious sister "Introduction" claims our notice.

This sententious dame, apparently o'er-learned in quotations, observes of botany, that "it is a science which has been cultivated by the wisest of mankind, and particularly by the professors of the medical art." She is, however, scarcely intelligible in the truism that, "notwithstanding the importance of this science *in* a branch of medical knowledge, it is much to be regretted that it is *so little* attended to by gentlemen of the faculty in this country. Indeed, *so much so*, that it obliges them to depend on the pretended skill of the ignorant and illiterate for many of their efficacious officinal plants, frequently at the expense of their own character, and of all that is valuable to their patients:" and her memory seems but bad, for she twice inserts the same quotation of eight long lines in the course of a few short pages, (vide pp. xi. and xix.;) doubtless by her *imperverted* mind "having in this occupation the violent passions lulled into a dead calm," which seems to be cherished as "an *unalloyed pleasure*."

But we now approach the shrine to which these "*vaunt* couriers" have led the way, and must assume a serious strain, difficult as it may be, among the numerous excitements to mirth with which the way seems thronged.

The object of the "First Lines of Botany" being professedly "to initiate the young botanist, and such as have no distinct botanical ideas, to the general study of this useful and pleasing science," we might reasonably expect, if not a profound treatise, at least a correct exposition of common principles; if not an original essay, at least a perspicuous compilation of known facts; and this more especially in a work that "attempts to correct the want of method, in an elementary point of view, by tracing 'the First Lines of Botany' in a simplified and systematic manner, in order to facilitate the introduction to a general knowledge of the science, through the medium of an agreeable and instructive series of connexions, leading the student, link by link, almost imperceptibly, to the acquisition of his object, which embraces a general acquaintance with the vegetable kingdom, the clear understanding of all botanical writers and systems, but more especially that of Linnæus." Grievous disappointments would follow any such expectations. But we err: there is much that is original in this book, more than is often found in the same number of pages, whether we regard the construction of the sentences, the orthography of the words, or the ideas which they propound; many of which are, and ever will be, peculiar to the author. Let a few specimens, both of the matter and the manner, suffice for illustration: we will append our observations as a running commentary on the extracts that we make.

The book lies open at page 89, and we there read, "As regards *submarine vegetables*, namely, as those which grow in and under the sea, as the large green membranous sea belts, which grow on stones; the fuci and other sea-weeds; the *corallines* on stones and oyster-shells; the *sea fan*; the *coral*, which grows on rocks; the sponge, &c." Has the author slept since the time of Ray, that corals and corallines are to be classed as *vegetables?* Has Ellis written in vain? and has even the favorite Linnæan system been forgotten?

Again, on the same page, under the head Poisonous Mushrooms, we read, "Among the numerous species of fungous plants, none are so decidedly poisonous as the pepper agaric, the deadly agaric, *champignon*, and some others." Strange classification this! for the champignon (agaricus pratensis) is a dietetic plant, and one of the least noxious of the entire order.

In a preceding page (86) we read, "Mushrooms, moss, and *other fungous excrescences*, are a spurious kind of plants, or rather excrementitious plants, since they *entirely* arise from the bodies of other plants, or from a kind of viscous mucilage of the earth. They indeed grow, and have roots, some inserted into the fibres of the plant producing them; e. g. misletoe is radicated into the fibres of the oak, and moss to the fibres of the bark of trees; mushrooms arise from various matters in earth and wood," &c. Most unintelligible confusion! Moss is *not* a fungous excrescence; neither are mushrooms excrescences, in the correct and ordinary acceptation of the term; neither do they arise entirely from the bodies of other plants, or from a viscous mucilage of the earth; and why the misletoe is associated with moss and mushrooms, we know not.

Page 25: "Botanical writers mention three kinds of *bulbous* roots, viz. the solid, or those of one solid substance, as the *turnip*." The root of the turnip is not a bulb, any more than that of the carrot or the parsnip: indeed, neither of them have the essential character, and the botanical term has been long restrained to such roots only as bear hybernacles or subterranean buds: the colchicum or crocus should have been cited as examples.

Page 31: "The *fibrils* are apparently a production of the rootlets, with a few ligneous vessels shooting into their centre. Whatever may be their structure, their pores are evidently the absorbent mouths of the root, possessing either a valvular apparatus or a power of contracting strongly." Gratuitous assumptions! the imbibition and rise of the sap depend on a very different cause, viz. electro-filtration.

Page 71: "The motion of the sap *is* as follows: 1. The nutritious sap ascends, the first year of a plant's growth, by the vessels of the pith; after which the pith becomes dry, and so continues. 2. The next part through which the sap rises, is the wood, by the air-vessels, and that only in spring. 3. The third part by which it ascends is the bark, as already stated, the greatest part of the year; and this *is* the general theory of the motion of the sap." Such *was* formerly, but such *is not now* the general theory of the motion of the sap. A more modern, yet still imperfect, physiology is that slightly noticed at page 174. The subject is one of much interest, but we dare not digress.

Much as we value the works of Nehemiah Grew, and great as we consider his authority, still to propound, in a simplified introduction to physiological botany, speculations read to the Royal Society about 1670, and exploded upwards of a century ago, when more accurate and more extended observations led to the formation of a more just and reasonable doctrine, does seem to us, to use our author's words, "as ridiculous as absurd." The same objection holds against the use of many terms long since obsolete; and, if we tolerate "*empalement*" for the calyx, and *apices* for the anthers, we cannot "*attire*" for the stamina and pistilla, which was at first erroneously applied, nor "*foliage*" or "*leaves*" for the petals and corolla; as such terminology cannot fail to re-induce that confusion from which the science has so long striven to emerge. The same observations apply to many other parts, vide pages 83 and 84, &c.

It is useless to proceed with this analysis: one or two more examples, and we have done.

Pages 101 and 102: The definitions of the orders of the class Syngenesia, are both insufficient and incorrect.

P. 111: "Class x. Decandria. In this class are comprised several trees of foreign growth; also the *fly-trap of Venus*, a native of America."

P. 125: "The American plant called *dionœa muscipula*, or *Venus' fly-trap*, affords another instance of vegetable motion. This plant is placed, according to the Linnæan, or sexual system of botany, in the class Diadelphia, two brotherhoods, ten males. (See p. 114.)" How are these two statements to be reconciled? In the description of this plant, p. 125, the natural history of the campion is confused with that of the muscipula: and this the quotation from the Botanic Garden sufficiently exemplifies.

In the first chapter of Part iii., after enumerating many of the most important and curious of the vital phenomena evinced by plants, such as their waking and their sleep, their motions, their selection of food, their delicate irritability, &c., the author observes, "But facts of this kind, however they may excite our wonder, are *far from* proving that *vegetables live!*" What, in the name of botany, do they prove? not the sensation or perception of plants, we will agree with our author, but most incontestibly their *life*.

We like to be useful, as far as in our power lies, and will therefore add a glossary of some expressions in the volume, which are certainly hard to be understood; for, after reference to the page of corrigenda, and making every liberal allowance for the carelessness of the printer's devil, who seems to have been a reckless fellow, we freely confess that we often were at fault, and not seldom fairly stumbled: e. g. page 106, Class iii. Triandria. "The general character of grasses, of which there are upwards of 300 species, may be described as follows: the leaves furnish pasturage for cattle, the smaller seeds serve as food for birds, and the larger for man; some, however, are preferred to others, as *fescue* for sheep, *meadow grass* for cows, *canary* for small birds, *oats* and *beans* for horses, *rye, wheat*, and *barley* for man." We certainly knew that horses ate oats and beans, but never before dreamt that the bean was a species of grass, or should have guessed that the *natural history* of these, or any other plants, would have been given as their *general character*. But enough; and now the glossary.

P. 8. "The *advantages* resulting from a knowledge of botany *is* (*are?*) self-evident. Do., do., do., *passim*.

12. "The waving corn and golden *sheaths*, (sheaves?)

27. " The branches or *bows*, (boughs?)
32. " The stem or *stone*, (stalk?)
39. " The straw of *grapes*, (grasses?)
60, 67. " Stamina, Parenchyma, obsolete definitions.
103, 104, 107, 108, and 109, &c. "*calx*," (calyx?)
114, 124. " *Heydasarum*, (Hedysarum?)

144. "To inosculate, means *anatomically* the forming of the mouths of the capillary veins and arteries." When we were in the schools, it used to signify the interunion of vessels, and not the forming of mouths.

169. "Oxygen is derived from οξυς, acid, and μεννάϖ, I generate." We used to write these words, ὀξὺς and γεννάω. Lavoisier, who coined the term oxygen, derived it from the passive, γείνομαι.

But we are weary, and we find that our glossary would almost extend to a verbal index of the work.

In the long dissertation on Dew, how comes it that the old works of Dufay and Muschenbrock are cited, to the neglect of the more recent and less exceptionable experiments of Mr. Daniell and Dr. Wells? But this antiquarian predilection pervades the volume; and the last fifty pages contain so much poetry and prose run mad, as to defy all sober criticism. We quit it in despair.

